# Gastro-Intestinal Catarrh of the Ox1Read before the Illinois State Veterinary Medical Association, Champaign, Illinois, February 17, 1903.

**Published:** 1903-05

**Authors:** F. H. Barr

**Affiliations:** Pana, Ill.


### GASTRO-INTESTINAL CATARRH OF THE OX.[1]

By Dr. F. H. Barr,
PANA, ILL.

This is a disease with which we are more or less familiar, especially those of us who have to do with practice among dairy cattle. It is not my purpose to shed any new light on this trouble, but rather to give a description of the disease as I have found it, in a hope that I may succeed in bringing out a liberal discussion on the subject which to the rural practitioner is of great importance.

First, we will consider the etiology.

All animals are not equally predisposed to gastro-intestinal catarrh or digestive troubles of any kind. This predisposition varies according to the degree of activity of the digestive function, the constitution and temperament, pre-existing disease, age, etc.

In ruminants diseases of the stomach are more common than of the intestines. The rumen and omasum, first and third stomachs, are the gastric compartments most frequently affected.

The principal cause predisposing animals to disease is a continued feeding of poor, insipid food and slops, such as mouldy hay or rotten corn, and sour, fermented swill, or a diet composed entirely of liquids to the exclusion of all other, and we might add that poor stabling in narrow stalls, as I have frequently found it, where but one or two cows are kept in a little cramped-up shed, and fed on all manner of refuse.

We always find that the trouble is more common in the seasons of spring and fall.

The causes that develop it are, 1st, overloading the rumen; 2d,

[1] Read before the Illinois State Veterinary Medical Association, Champaign, Illinois, February 17, 1903.

frosted or frost-covered grass or fodder, feeding on pastures before the grass is sufficiently mature in the spring.

It is likely that animals affected with gastro-intestinal catarrh undergo a period of indigestion or dyspepsia manifested by not masticating the food sufficiently the second time, an abbreviated motion of the paunch, etc., but this is a period when the owner does not notice that the animal's condition is other than normal. Closely following this condition we have gastro-intestinal catarrh.

First, in the acute form; later, if not properly treated, it has been my observation we either have the chronic form or death.

Semeiology. The practitioner is called to see we will say a dairy cow, and the owner tells us that the cow seemed constipated, wouldn't eat much, stood in the stall with her back arched, and that at the last milking she didn't give any milk; or he may say that she is very loose, instead of being constipated, as the case may be, and upon our arrival we find that fact true, but he has neglected to tell us that owing to the fact that she had lost her cud. He had given her a greasy dish-rag, a pound of fat bacon covered with coal soot, and neither had enabled her to find it, and that he had given her two pounds of Epsom salts. Well, to make his usually long story short, we find an animal in an extremely bad condition, manifesting all the symptoms of acute gastro-intestinal catarrh, which is as follows:

The animal keeps away from the manger; the spinal column is arched; the limbs drawn together; the hair is dull and bristly, especially about the neck; the ears hang pendant; the cutaneous temperature is variable; ears and horns are alternately hot or cold; the muzzle is damp in some patients; others, it is dry. If damp, and the moisture is wiped off, it will be slow to return; the mouth is hot and is sometimes full of ropy saliva; the appetite is depressed or entirely gone. Regurgitations are stopped or, if at all, very seldom, and in small bolus and mastication is but slight until the food is again swallowed.

Sometimes we find vomiting. This, however, is seldom the case. The distended paunch is usually visible over left side, and the cheese-like contents can be felt through the flank. Occasionally I have found the stomach distended with gas. The paunch never undergoes normal rotation as in health, the motion is slight.

Defecation is rare, and may be ring-streaked or covered with mucus. When diarrhœa is present, on examination we find alimentary material that has never undergone the second or merycismastication; the matter usually has a very offensive smell.

The animal frequently wears an anxious look, with trembling in the flanks, looks toward the flanks. The temperature is variable, but I have seldom ever been able to detect other than an average normal temperature. The pulse, like that of temperature, is variable, but usually accelerated—ten to twenty beats per minute. The lacteal secretion is usually reduced one-half to two-thirds.

Mild cases in the acute form frequently recover. Improvement is first noticed by the movement of the paunch becoming more frequent. Rumination gradually reappears, lactation improves, and, in fact, all actions become more nearly normal as the condition improves.

TREATMENT. Always enforce an almost complete restriction from food as long as rumination is suspended, but occasionally tempt the animal with a handful of loose hay or well-matured grass. This restriction should be maintained for several days even after rumination is again established. Always try to have animals partake of as much water as possible; keep in pleasant, comfortable quarters. I have found friction or massage of the abdomen of benefit.

Medical agents which I have employed with greatest success have been: Glauber salts, with gum acacia in doses of four to five ounces of the salts to one ounce of the gum acacia to one quart of water, given at intervals of three to four hours, to increase peristalsis. Twice a day injection of eserine sulphate in one-grain doses. In my experience I consider that the enforcement of a strict diet with plenty of water with temperaturc lowered of as much value as medicinal agents.

